# Determination of the Volatile Composition in Brown Millet, Milled Millet and Millet Bran by Gas Chromatography/Mass Spectrometry 

**DOI:** 10.3390/molecules17032271

**Published:** 2012-02-24

**Authors:** Jingke Liu, Xia Tang, Yuzong Zhang, Wei Zhao

**Affiliations:** 1 Institute Millet Crops of Heibei, Academy of Agriculture and Forestry, No.162, Hengshan St., Shijiazhuang, Hebei 050035, China; 2 National Millet Improvement Center of China, No.162, Hengshan St., Shijiazhuang, Hebei 050035, China; 3 Minor Cereal Crops Research Laboratory of Hebei Province, No.162, Hengshan St., Shijiazhuang, Hebei 050035, China; 4 College of Food Science and Technology, Agricultural University of Hebei, No.2596, Lekainan St., Baoding, Hebei 071001, China

**Keywords:** volatiles, brown millet, milled millet, millet bran, GC/MS

## Abstract

The volatile compounds from brown millet (BM), milled millet (MM) and millet bran (MB) were extracted using simultaneous distillation/extraction with a Likens-Nickerson apparatus. The extracts were analysed using gas chromatography coupled with mass spectrometry (GC-MS). A total of 65 volatile compounds were identified in all of the samples. Among these compounds, 51, 51 and 49 belonged to BM, MM and MB, respectively. Aldehydes and benzene derivatives were the most numerous among all of the compounds. Three compounds (hexanal, hexadecanoic acid and 2-methylnaphthalene) were dominant in the BM and MM materials. Eight compounds (hexanal, nonanal, (*E*)-2-nonenal, naphthalene, 2-methylnaphthalene, 1-methylnaphthalene, hexadecanoic acid and 2-pentylfuran) were dominant in the MB materials. Apart from the aromatic molecules, which were present in all fractions, compounds present only in BM, MM or MB were also identified.

## 1. Introduction

Foxtail millet (*Setaria italica* Beauv) is one of the oldest cultivated cereals, which has its origins in China and subsequently was extended into India and over most of Africa and parts of the southern United States [1]. Foxtail millet plays a very important role in the agriculture and food industry of many developing countries because of its capacity to grow under adverse heat and limited rainfall conditions. This millet is rich in carbohydrates [2,3,4] and protein [5,6] and also contains oils [7,8] and vitamins [9]. It has been recorded that foxtail millet has many nutritional and medical functions. It has been used in China as a Traditional Medicine to invigorate the stomach, strengthen the spleen, quench thirst and promote urination. In some cases, this millet can also be used in the treatment of diabetes and cardiovascular diseases [10].

In recent years, increasingly more foxtail millet products have entered into the daily lives of people, including millet porridge, millet wine, and millet nutrition powder. In addition to its nutritional benefits, the unique odour of the millet is one of the main reasons why consumers prefer it. Odour is considered a critical quality trait in cereals; it affects consumer preference because it travels to the nose during consumption and stimulates the olfactory receptors in the nasal cavity [11]. The aromatic characteristics of various cereals, such as rice [12,13], corn [14,15,16], wheat [17,18,19] and buckwheat [20,21,22,23], have been investigated from the perspective of the volatile compounds they contain. Aldehydes, alcohols, ketones, benzene derivatives, hydrocarbons, acids, esters, heterocycles and sulphur-containing compounds are the main volatile compounds of these cereals. Among them, odour active compounds of rice were analysed in detail, and 2-acetyl-1-pyrroline was reported as the most important odour active compounds contributing to the odour of popcorn [12,13]. Although the volatile constituents of the grain are believed to play a major role in cereal quality, relatively little information is available concerning these components in foxtail millet.

Dehusking and polishing are important steps in grain processing. These steps not only improve the sensory and edible quality of grain but also yield the main product and by-products, including brown grain, milled grain and bran. Compared with milled grain, brown grain is a nutritionally valuable food. The bran layers of brown grain kernels are rich in dietary fibre, minerals, oil, protein and vitamins (particularly B vitamins) [24,25,26,27]. When added to the diet, bran layers are effective in reducing cholesterol levels in humans [28,29,30]. The utilisation of brown grain and its bran has gradually become one of the main directions of development in diet adjustments. However, most of these studies have focused on nutrition but have ignored differences in the volatile compounds.

To conduct a thorough investigation into the volatile flavours of foxtail millet and its foment its increased utilisation, an evaluation of the differences in volatile compounds among brown millet (BM), milled millet (MM) and millet bran (MB) is essential. Therefore, the main objective of this research was to identify and compare the volatile components of BM, MM and MB using simultaneous distillation extraction (SDE) and gas chromatography-mass spectrometry (GC-MS).

## 2. Results and Discussion

In this study, the volatile compounds from brown millet, milled millet and millet bran were extracted using the simultaneous distillation method and detected using GC and GC-MS. The experimental results are provided in Table 1. A total of 65 volatile compounds were tentatively identified. These compounds included aldehydes (17 compounds), alcohols (five compounds), ketones (nine compounds), hydrocarbons (seven compounds), acids (three compounds), benzene derivatives (12 compounds), esters (four compounds), furans (five compounds) and S-containing compounds (three compounds). In total, 52 compounds were identified: 51 in BM, 51 in MM, and 49 in MB. Aldehydes and benzene-containing compounds were the most numerous among all the compounds. The major volatile components present in BM, MM and MB were hexanal (12.82 ± 0.42%), hexadecanoic acid (16.43 ± 1.28%) and 2-methylnaphthalene (8.87 ± 0.54%); hexanal (7.49 ± 0.42%), hexadecanoic acid (35.77 ± 1.62%), 2-methylnaphthalene (5.36 ± 0.21%) and hexanal (7.69 ± 0.27%); and nonanal (5.89 ± 0.17%), (E)-2-nonenal (5.62 ± 0.18%), naphthalene (6.75 ± 0.34%), 2-methylnaphthalene (14.18 ± 0.26%), 1-methylnaphthalene (5.61 ± 0.39%), hexadecanoic acid (8.98 ± 0.29%) and 2-pentylfuran (5.11 ± 0.18%), respectively.

**Table 1:** Volatile components identified in BM, MM and MB.

RI	Compound name		Peak area (%) ± SD				Identification
			BM		MM	MB	
	Aldehydes						
803	Hexanal		12.82 ± 0.42		7.49 ± 0.42	7.69 ± 0.27	MS,RT
848	( *E*)-2-Hexenal		0.22 ± 0.03		0.11 ± 0.01	0.23 ± 0.02	MS,RT
903	Heptanal		2.01 ± 0.07		0.75 ± 0.11	2.90 ± 0.09	MS,RT
953	( *E*)-2-Heptenal		2.47 ± 0.03		1.29 ± 0.04	n.d.	MS,RT
1002	Octanal		0.44 ± 0.03		0.19 ± 0.01	0.44 ± 0.06	MS,RT
1016	( *E,E*)-2,4-Heptadienal		0.24 ± 0.02		0.12 ± 0.02	0.16 ± 0.03	MS,RT
1045	Benzeneacetaldehyde		0.25 ± 0.01		0.96 ± 0.05	2.03 ± 0.09	MS,RI
1057	( *E*)-2-Octenal		1.54 ± 0.07		0.91 ± 0.17	1.55 ± 0.05	MS,RT
1106	Nonanal		3.50 ± 0.23		2.23 ± 0.13	5.89 ± 0.17	MS,RT
1163	( *E*)-2-Nonenal		1.84 ± 0.14		2.63 ± 0.16	5.62 ± 0.18	MS,RT
1208	Decanal		0.53 ± 0.02		0.46 ± 0.04	1.06 ± 0.10	MS,RT
1218	( *E,E*)-2,4-Nonadienal		0.46 ± 0.02		0.35 ± 0.03	0.42 ± 0.02	MS,RT
1312	( *E,E*)-2,4-Decadienal		4.43 ± 0.48		3.36 ± 0.17	2.49 ± 0.36	MS,RT
1409	Dodecanal		n.d.		0.05 ± 0.01	n.d.	MS,RT
1509	Tridecanal		0.37 ± 0.04		n.d.	0.27 ± 0.02	MS,RT
1614	Tetradecanal		0.65 ± 0.09		n.d.	n.d.	MS,RT
1716	Pentadecanal		2.43 ± 0.14		0.87 ± 0.06	0.93 ± 0.05	MS,RT
	Subtotal		34.21 ± 0.96		21.76 ± 1.05	31.65 ± 0.20	
	Benzene derivatives						
855	Ethyl-benzene		n.d.		0.03 ± 0.00	n.d.	MS,RT
868	1,3-Dimethylbenzene		0.18 ± 0.02		0.07 ± 0.00	n.d.	MS,RI
1114	1,2,4,5-Tetramethylbenzene		n.d.		n.d.	0.19 ± 0.01	MS,RI
1179	Naphthalene		4.93 ± 0.26		2.35 ± 0.15	6.75 ± 0.34	MS,RT
1284	2-Methylnaphthalene		8.87 ± 0.54		5.36 ± 0.21	14.18 ± 0.26	MS,RI
1305	1-Methylnaphthalene		3.64 ± 0.28		2.30 ± 0.05	5.61 ± 0.39	MS,RT
1351	Biphenyl		0.83 ± 0.04		0.14 ± 0.01	4.95 ± 0.58	MS,RI
1366	1-Ethylnaphthalene		1.58 ± 0.08		2.30 ± 0.07	n.d.	MS,RT
1426	1,6-Dimethylnaphthalene		n.d.		2.37 ± 0.07	n.d.	MS,RI
1436	Diphenylmethane		0.36 ± 0.02		0.24 ± 0.00	0.46 ± 0.05	MS,RI
1566	Fluorene		0.69 ± 0.03		0.35 ± 0.02	0.51 ± 0.02	MS,RI
1794	Phenanthrene		0.59 ± 0.16		0.39 ± 0.01	0.29 ± 0.03	MS,RI
	Subtotal		21.67 ± 0.76		15.90 ± 0.30	32.94 ± 1.57	
	Alcohols						
766	1-Pentanol		1.16 ± 0.11		n.d.	0.31 ± 0.03	MS,RT
872	1-Hexanol		2.41 ± 0.13		0.86 ± 0.09	1.42 ± 0.03	MS,RT
982	1-Octen-3-ol		0.28 ± 0.03		0.46 ± 0.05	0.98 ± 0.12	MS,RT
1069	( *Z*)-2-Octen-1-ol		n.d.		n.d.	0.38 ± 0.02	MS,RI
1079	1-Octanol		0.21 ± 0.01		n.d.	0.14 ± 0.02	MS,RT
	Subtotal		4.06 ± 0.20		1.31 ± 0.14	3.23 ± 0.10	
	Ketones						
891	2-Heptanone		1.03 ± 0.05		0.38 ± 0.03	0.26 ± 0.01	MS,RT
974	1-Octen-3-one		n.d.		n.d.	0.29 ± 0.02	MS,RI
983	2,5-Octanedione		0.19 ± 0.03		0.07 ± 0.02	n.d.	MS,RI
1036	3-Octen-2-one		0.30 ± 0.01		n.d.	n.d.	MS,RT
1065	Acetophenone		0.14 ± 0.01		0.06 ± 0.01	0.14 ± 0.01	MS,RI
1086	( *E,E*)-3,5-Octadien-2-one		0.75 ± 0.02		0.59 ± 0.03	0.08 ± 0.01	MS,RI
1135	3-Nonen-2-one		0.08 ± 0.01		0.04 ± 0.00	0.04 ± 0.00	MS,RT
1859	2-Heptadecanone		0.60 ± 0.05		n.d.	n.d.	MS,RI
1921	( *E,E*)-6,10,14-Trimethyl-,9,13-		1.73 ± 0.11		0.50 ± 0.04	n.d.	MS,RI
	pentadecatrien-2-one						
	Subtotal		4.82 ± 0.05		1.63 ± 0.11	0.81 ± 0.03	
	Hydrocarbons						
1200	Dodecane		0.88 ± 0.04		n.d.	n.d.	MS,RT
1300	Tridecane		0.12 ± 0.01		0.09 ± 0.01	n.d.	MS,RT
1500	Pentadecane		1.02 ± 0.12		0.88 ± 0.05	1.77 ± 0.21	MS,RT
1600	Hexadecane		1.75 ± 0.15		1.42 ± 0.08	2.28 ± 0.13	MS,RT
1700	Heptadecane		0.74 ± 0.22		0.92 ± 0.04	n.d.	MS,RT
1800	Octadecane		0.39 ± 0.08		0.69 ± 0.03	0.58 ± 0.03	MS,RT
1900	Nonadecane		n.d.		0.87 ± 0.04	0.32 ± 0.03	MS,RT
	Subtotal		4.90 ± 0.37		4.86 ± 0.10	4.95 ± 0.35	
	Acids						
1765	Tetradecanoic acid		0.29 ± 0.01		0.31 ± 0.03	n.d.	MS,RT
1833	Pentadecanoic acid		n.d.		0.47 ± 0.04	0.37 ± 0.01	MS,RT
1951	Hexadecanoic acid		16.43 ± 1.28		35.77 ± 1.62	8.98 ± 0.29	MS,RT
	Subtotal		16.72 ± 1.29		36.54 ± 1.65	9.35 ± 0.29	
	Esters						
1264	Propyl benzoate		n.d.		0.16 ± 0.01	0.17 ± 0.01	MS,RI
1334	Benzoic acid, butyl ester		0.81 ± 0.07		0.21 ± 0.00	1.76 ± 0.14	MS,RI
1936	Hexadecanoic acid, methyl ester		n.d.		0.24 ± 0.02	n.d.	MS,RT
1968	Hexadecanoic acid, ethyl ester		0.98 ± 0.05		n.d.	0.24 ± 0.03	MS,RI
	Subtotal		1.79 ± 0.09		0.61 ± 0.03	2.18 ± 0.17	
	Heterocycles						
812	3-Furaldehyde		n.d.		n.d.	0.27 ± 0.03	MS,RI
829	Furfural		n.d.		0.04 ± 0.00	0.05 ± 0.01	MS,RI
865	2-Furanmethanol		n.d.		n.d.	0.13 ± 0.01	MS,RI
907	1-(2-Furanyl)-ethanone		n.d.		n.d.	0.27 ± 0.01	MS,RI
992	2-Pentylfuran		3.73 ± 0.08		2.94 ± 0.14	5.11 ± 0.18	MS,RT
	Subtotal		3.73 ± 0.08		2.98 ± 0.13	5.83 ± 0.21	
	Sulphur-containing						
916	Diethyl disulphide		0.11 ± 0.02		0.04 ± 0.01	0.04 ± 0.01	MS,RT
1025	2-Acetylthiazole		0.13 ± 0.01		0.08 ± 0.01	0.28 ± 0.05	MS,RI
1220	Benzothiazole		0.09 ± 0.01		0.32 ± 0.00	0.43 ± 0.04	MS,RI
	Subtotal		0.33 ± 0.02		0.44 ± 0.01	0.74 ± 0.05	
	Total		92.32 ± 4.82		86.03 ± 3.25	91.68 ± 2.97	

MS: confirmed by mass spectrum analysis; RT: identified by retention time of standard compounds; RI: identified by retention index; n.d.: not detected.

### 2.1. Aldehydes

Aldehydes represented the largest group in the number of volatile components identified in all of the samples, comprising 21.81–34.21% of the total volatile compounds detected. The composition of aldehydes differed both qualitatively and quantitatively among the three samples. The volatile fraction was mainly composed of hexanal, nonanal and (*E*)-2-nonenal; the three aldehydes had concentrations of >5% (and existed in at least one of the samples), and hexanal was the most abundant aldehyde (>7%). BM, MM and MB contained 16, 15 and 13 aldehydes, respectively. (*E,E*)-2,6-Nonadienal, tridecanal and (*E*)-heptenal were absent in BM, MM and MB, respectively. Tetradecanal was unique to BM, whereas dodecanal was unique to MM. Aldehydes usually are derived from the autoxidation and enzymolysis oxidation of the double carbon-carbon bond of unsaturated fatty acids present in cereals [31,32,33]. In general, aldehydes have a great impact on the aroma of a cereal product because of their low odour threshold values. Aldehydes detected in the three samples belonged to the *n*-alkanal, 2-alkenal, and 2,4-alkadienal classes, respectively. The *n*-alkanals from hexanal to decanal are aromatics and provide grassy, green and fatty (hexanal), fatty and citrus (heptanal), fatty, soapy and green (octanal), fatty, citrus and green (nonanal) and fatty and grass (decanal) characteristics [31,32,33]. In addition, C_13_–C_18_ aldehydes of low concentrations were expressed in the samples; these long-chain aldehydes typically have high thresholds [33]; therefore, they may contribute little to the flavours of the samples. The 2-alkenals and 2,4-alkadienals have very low odour thresholds, which is the reason they are prevalent in cereal in small quantities. (*E*)-2-Nonenal, (*E*)-2-octenal, (*E*)-2-heptenal, (*E*)-2-hexenal belong to the 2-alkenals; with increasing C-chain length, the odour becomes less citrusy and fruity and more fat-like, and the odour threshold decreases [34]. In terms of the 2,4-alkadienals, (*E,E*)-2,4-nonadienal and (*E,E*)-2,4-decadienal have been reported as the key odorants in rice, responsible for the “nutty and fatty” and “fatty” characteristics [35,36]. Abundant aldehydes detected in all of the samples are very important in the odour of the millet.

### 2.2. Benzene Derivatives

Benzene derivatives were the second largest group identified in the samples. In BM, MM and MB, nine, nine and 11 benzene derivatives were detected, respectively. From highest to lowest, the benzene derivative contents were: 32.94% in MB, 21.67% in BM and 15.90% in MM. Naphthalene, 2-methyl-naphthalene, 1-methylnaphthalene, and 1-ethylnaphthalene were present in concentrations of >5% (and existed in at least one of the samples). Ethylbenzene and 1,6-dimethylnaphthalene were only present in MM, and 1,2,4,5-tetramethylbenzene was only detected in MB. The compounds 1,3-dimethylbenzene and 1-ethylnaphthalene were missing in MB. Among benzene derivatives, naphthalene, 2-methylnaphthalene, 1-methylnaphthalene, biphenyl, 1-ethylnaphthalene, 1,6-dimethyl-naphthalene, fluorene and phenanthrene, which belong to polycyclic aromatic hydrocarbon (PAH), should be noted. Recent epidemiological studies have revealed that dietary exposure to PAHs is associated with an increased risk of some human cancers [37]. These compounds possibly come from environment contamination including air, soil and water, in which they are formed during the incomplete combustion of organic materials [38]. Benzene derivatives are typically closely related to the odour of cereal. Among the benzene derivatives detected, naphthalene, 2-methylnaphthalene, 1-methylnaphthalene, and 1-ethylnaphthalene have been identified as odour-active compounds in cereal; they are related to naphthalene aromatic aspects [35,36,39]. Compared with other cereal, such as rice and buckwheat, more benzene derivatives were identified in the millet. These compounds, especially the odour-active benzene derivatives, may contribute to the flavour of millet and distinguish it from other cereal. 

### 2.3. Alcohols and Ketones

The amounts and contents of alcohols and ketones detected in the samples were relatively small. In total, four, two and five alcohols were detected in BM, MM and MB. (*Z*)-2-Octen-1-ol was absent in BM and MM; the missing alcohols in MM were 1-pentanol and 1-octanol. Despite the small amounts of alcohols detected, most of these compounds were relevant to the odour of the cereal. Alcohols are generally formed by the decomposition of the secondary hydroperoxides of fatty acids [40]. They generally supply the fruity, floral and grassy aspects to cereal. Among the various alcohols detected, 1-pentanol, 1-hexanol, 1-octen-3-ol and 1-octanol have been identified as the odour-active compounds in rice and have fruity and plastic, green, mushroom and citrus aromatic characteristics, respectively [35,36,39,41]. 

In total, eight, six and five ketones were present in BM, MM, and MB. The compounds 2,5-octanedione and 2-heptadecanone and 1-octen-3-one were unique ketones in BM and MM, respectively. The missing ketones in MB were 2,5-octanedione and (*E,E*)-6,10,14-trimethyl-5,9,13-pentadecatrien-2-one. The autoxidation of fatty acids, particularly unsaturated fatty acids, has been proposed to form ketones [40]. Ketones typically provide the soapy and fruity characteristics of food. Among the various ketones detected, 2-heptanone (fruity) and 3-octen-2-one (rose) have been identified as odour-active compounds in rice [36]. Therefore, the contributions of these compounds to millet cannot be ignored. 

### 2.4. Hydrocarbons, Acids and Esters

A certain number of hydrocarbons, acids and esters were also identified in all of the samples. In total, six, six and four hydrocarbons, two, three and two acids, and two, three and three esters were detected in BM, MM and MB, respectively. Except for hexadecanoic acid, whose content was 8.98–35.77%, most of these compounds were present in small quantities. The hydrocarbons identified in the samples included a homologous series of n-hydrocarbons ranging from C_12_ to C_19_. Dodecane was absent in MM and MB. The other absent hydrocarbon in BM and MB were nonadecane and tridecane and heptadecane, respectively. These hydrocarbons could result from the decarboxylation and splitting of carbon-carbon chains of higher fatty acids [40]. Acids identified in the samples included a homologous series of *n*-acids ranging from C_14_ to C_16_. Among the detected acids, tetradecanoic acid and pentadecanoic acid were absent in MB and BM, respectively. Esters identified in the samples were benzene and hexadecanoic acid esters. Hexadecanoic acid methyl ester was only present in MM, and propyl benzoate and hexadecanoic acid ethyl ester were absent in BM and MM, respectively. Hydrocarbons, acids and esters, typically have relatively high flavour thresholds and likely have little contribution to the odour of the millet. However, some of them are present at high levels in the samples, particularly hexadecanoic acid, and may thus play roles in the overall flavour.

### 2.5. Heterocycles and Sulphur-Containing Compounds

Some heterocycles and sulphur-containing compounds were also detected in the samples. In addition to 2-pentylfuran, which was present in all of the samples, furfural was detected in MM and MB; other heterocycles were only detected in the bran sample. These compounds, such as 3-furaldehyde (almond) [42], furfural (sweet, woody and almond) [43], 2-furanmethanol (sweet and honey) [44] and 1-(2-furanyl)ethanone (sweet, almondy and nutty) [44], have been identified as odour active compounds in some cereals and derived products. In addition, 2-pentylfuran should be noted, as it was detected in all the samples. The compound 2-pentylfuran has a relatively low threshold and a vegetal aromatic note [45]. The characteristic aromatic notes of sulphur-containing compounds have been described as nutty, roasted and corn-like. Dimethyl sulphide and 2-acetylthiazole contribute a cooked corn and nutty aroma to food, respectively [46]. These compounds are less abundant in cereal and may play an important role in millet.

## 3. Experimental Section

### 3.1. Materials

Foxtail millet (*Setaria italica*), var. JIGU NO19, was obtained from the Institute of Millet Crops, Heibei Academy of Agriculture and Forestry of China, and was stored in a refrigerator at −18 °C until use. After dehusking, 10% of the BM was milled out using an experimental mill (JLMZJ, Shanghai, China), and then the MM and MB were collected. The water, protein, fat, ash and carbohydrate contents were analysed (Table 2). Water, protein, fat, ash, and carbohydrate content were estimated by standard AOAC Methods [47].

**Table 2:** General compositions of BM, MM and MB.

	BM	MM	MB
Water	10.80 ± 0.18	10.93 ± 0.46	9.07 ± 0.34
Protein	6.42 ± 0.35	5.06 ± 0.31	6.78 ± 0.19
Fat	5.07 ± 0.59	1.75 ± 0.10	5.65 ± 0.23
Ash	1.99 ± 0.01	0.64 ± 0.04	2.15 ± 0.18
Carbohydrate	75.72 ± 0.81	81.62 ± 0.74	76.35 ± 0.63

Unit is g/100g dry matter.

### 3.2. Reagents

The water used in the study was purified using a Millipore-Q system (Millipore Corp., Saint-Quentin, France). Hexanal (99.5%), (E)-2-hexenal (98.0%), heptanal (99.0%), octanal (99.5%), (*E,E*)-2,4-heptadienal (88.0%), nonanal (98.0%), (*E*)-2-nonenal (98.4%), decanal (96.0%), (*E,E*)-2,4-nonadienal (85%), (*E,E*)-2,4-decadienal (99.0%), dodecanal (95.5%), tridecanal (88.0%), tetradecanal (96.0%), pentadecanal (95.0%), ethylbenzene (99.5%), naphthalene (99.5%), 1-methylnaphthalene (99.5%), 1-ethylnaphthalene (99.0%), 2-decanone (99.5%), 1-pentanol (99.7%), 1-hexanol (99.0%), 1-octen-3-ol (98.0%), 1-octanol (99.5%), 2-heptanone (99.0%), 3-octen-2-one (98%), 3-nonen-2-one (96%), dodecane (99.5%), tridecane (99.5%), pentadecane (99.5%), hexadecane (99.5%), heptadecane (99.5%), octadecane (99.0%), nonadecane (99.0%), tetradecanoic acid (99.0%), pentadecanoic acid (99.0%), hexadecanoic acid (98.0%), hexadecanoic acid methyl ester (98.0%), 2-pentylfuran (98%) and diethyl disulphide (96.0%) were purchased from Dr. Ehrenstorfer GmbH (Augsburg, Germany), Fluka (Buchs, Switzerland), Sigma (St. Louis, MO, USA), and TCI (Tokyo, Japan).

### 3.3. Simultaneous Distillation Extraction (SDE)

The extraction was performed using a modified Likens-Nickerson SDE apparatus for 2 h. The sample (200 g) and distilled water (2000 mL) were added to a 5000-mL round-bottom flask. Diethyl ether (50 mL) was added to another 100 mL round-bottom flask. Both flasks were connected to the apparatus, and additional distilled water was added into the central arm. The flask containing diethyl ether was heated using a water bath at 40 °C, and the flask containing the millet sample and distilled water was heated to the boiling point using an electric heating jacket. After extraction, the distillate in the 100-mL flask was dried over anhydrous sodium sulphate (5 g), concentrated using vacuum rotary evaporation and stored in headspace vials. Extraction of each sample was performed in triplicate. The results are reported as the mean values of relative peak area percent ± SD (standard deviation).

### 3.4. Gas Chromatography-Mass Spectrometry (GC-MS)

GC-MS was performed using an HP 5975B quadrupole mass selective detector (Agilent Technologies, Palo Alto, CA, USA). The mass spectral ionisation temperature was set to 230 °C. The mass spectrometer was operated in the electron impact ionisation mode at a voltage of 70 eV. Mass spectra were taken over an *m/z* range of 30–400. The flow rate of the helium carrier gas on the DB-5 column (30 m × 0.25 mm ID, 0.25 μm film thickness, J&W Scientific, Folsom, CA, USA) was 1 mL/min. The analysis was performed in the splitless mode, and the injector temperature was 250 °C. The column was held at 40 °C for 3 min and then increased from 40 °C to 220 °C at a rate of 4 °C/min, held at 220 °C for 2 min, and finally increased to 230 °C at a rate of 8 °C/min and held for 3 min. 

### 3.5. Identification of Components

The volatile components were identified by comparing their mass spectra with the mass spectra from MS libraries (NIST 05, WILEY 7.0). When available, the MS identifications were confirmed by comparing the GC retention times of the analytes with those from pure standards. The linear retention indices (RI) of the compounds were calculated using a series of *n*-alkanes (C_8_–C_20_, Sigma-Aldrich, Germany) injected under the same conditions. When standard chemicals were not available, tentative identification was achieved by matching the mass spectra and RI. The results are provided in Table 1.

## 4. Conclusions

This work presents the first study on the volatile compound contents of BM, MM and MB. A total of 65 volatile compounds were identified. Aldehydes and benzene derivatives were the most numerous among all of the compounds. Differences were noted in the volatile compounds present in different samples. In total, 34 compounds were detected in the BM, MM and MB. Apart from the aromatic molecules present in all the samples, compounds that were present only in brown millet, milled millet or bran were also identified. Compared with BM and MM, more heterocycles were present in MB. These differences may result in the different overall aromas of BM, MM and MB. 
